# Supplementary material for: Tamoxifen Activates Transcription Factor EB and Triggers Protective Autophagy in Breast Cancer Cells by Inducing Lysosomal Calcium Release: A Gateway to the Onset of Endocrine Resistance
Source: Int J Mol Sci. 2023 Dec 29;25(1):458. doi: 10.3390/ijms25010458 (PMC10779225; doi:10.3390/ijms25010458)
Supplement: Supplementary file 1 [file ijms-25-00458-s001.zip › ijms-2775845-supplementary.pdf]

Supplementary Materials

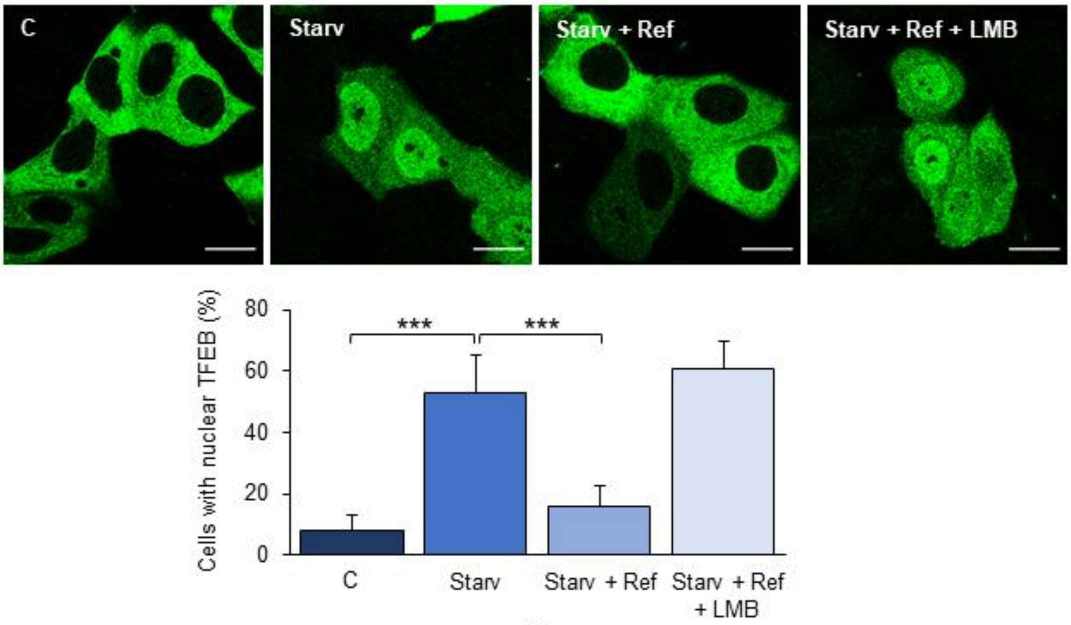

(a)

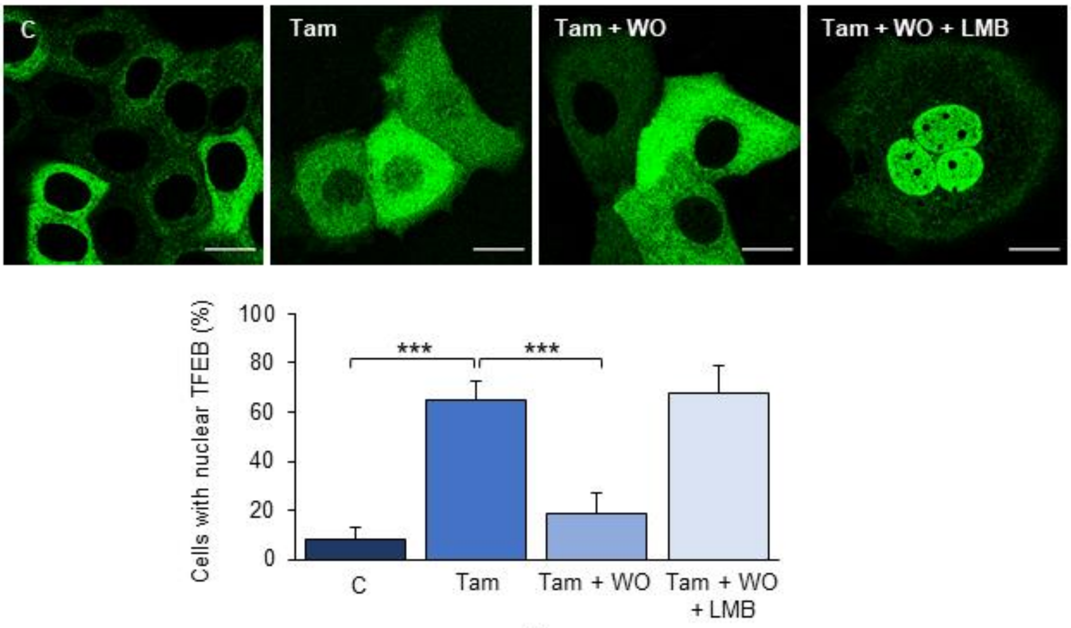

(b)

Figure S1. Effect of starvation or Tam on the subcellular localization of TFEB-GFP in MCF7 cells. (**a**) Cells starved for 4 h in HBSS-glucose and subsequently refed for 2 h with complete growth medium in the absence or presence of 10 ng/ml of leptomycin B. (**b**) Cells treated for 24 h with 5 µM Tam and after Tam removal (washout) either in the absence or presence of 10 ng/ml of leptomycin B. The bar charts below each set of images represent the percentage of cells with nuclear TFEB. C: controls; Starv: starvation; Ref: refeeding; LMB: 10 ng/ml leptomycin B; Tam: 5 µM tamoxifen; WO: washout. Data represent the mean ± SD of three independent experiments. Statistical significance was assessed by ANOVA followed by the Dunnett's *post-hoc* test; ***: $p < 0.001$. Scale bar: 20 µm

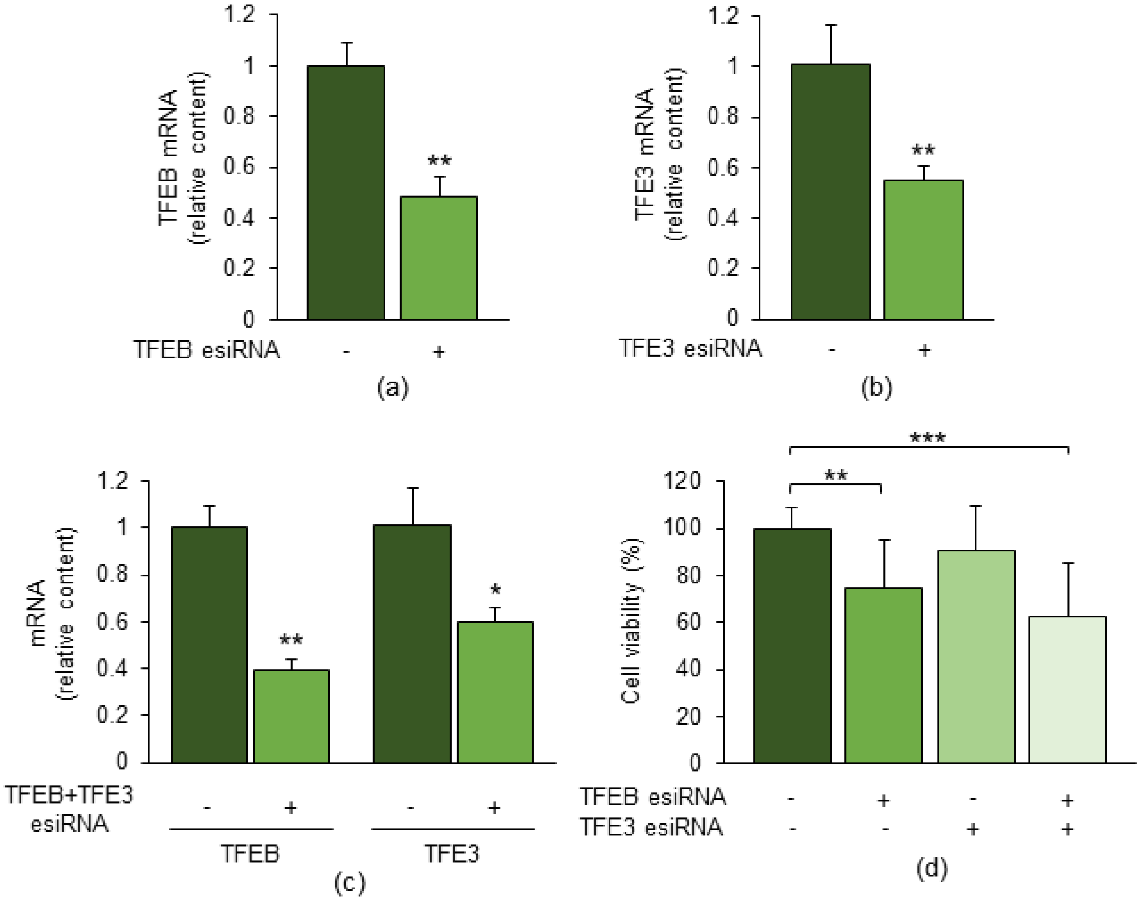

Figure S2. Effect of silencing TFEB, TFE3 or both on the viability of MCF7-TamR cells. Content of TFEB (**a**) or TFE3 (**b**) mRNA after 72 h of treatment of the cells with the indicated esiRNAs. (**c**) Effect of simultaneous silencing with siRNAs specific for TFEB and TFE3 on the mRNA amount of each target. (**d**) Effect of TFEB and/or TFE3 silencing on the viability of MCF7-TamR cells treated with 5 µM Tam for 72 h. Data represent the mean ± SD of three independent experiments. Statistical significance was assessed with the Student's *t*-test (panels **a**-**c**) or by ANOVA followed by the Dunnett's *post-hoc* test (**d**); **: $p < 0.01$; ***: $p < 0.001$

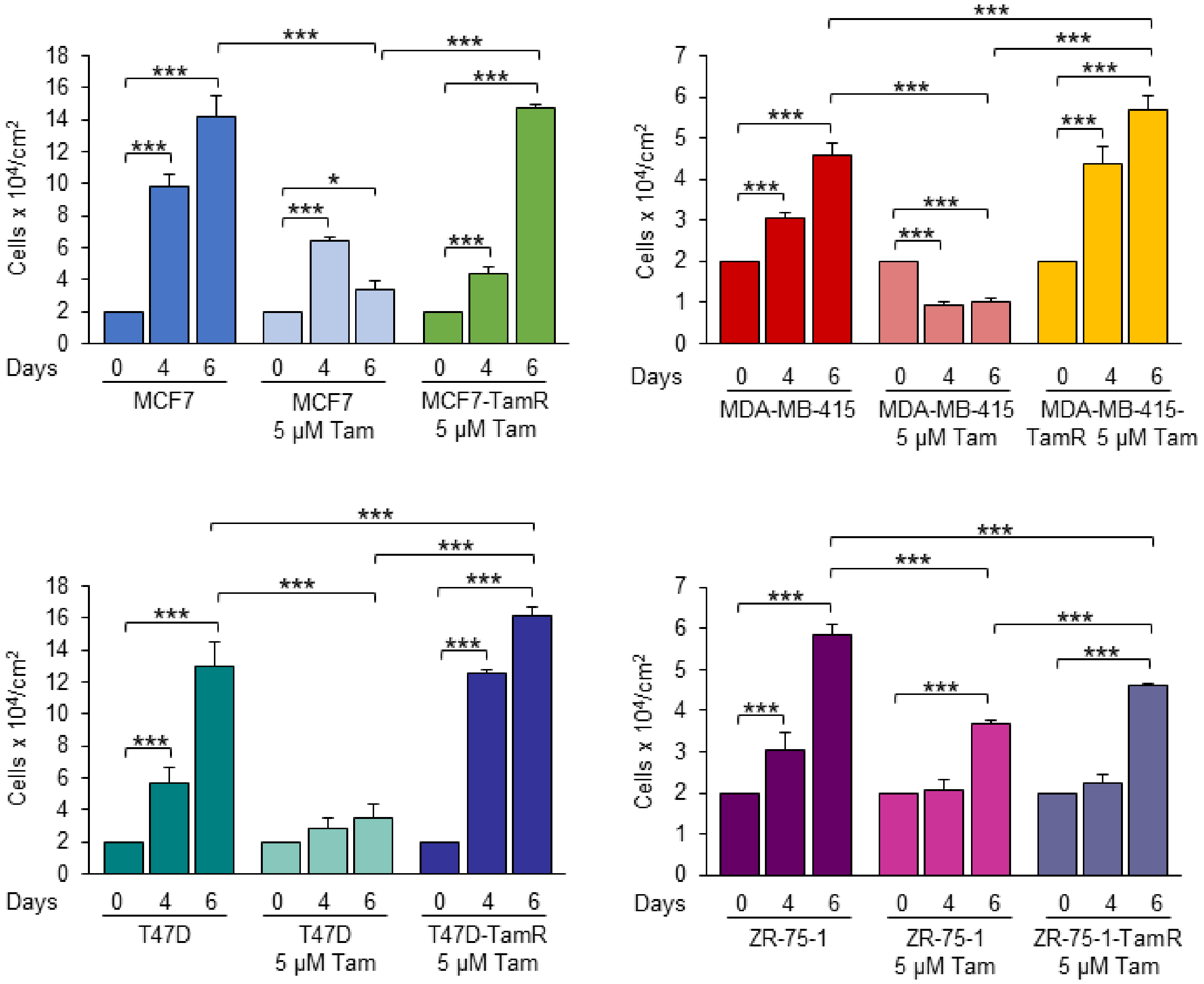

Figure S3. Growth of luminal A parental and of their corresponding Tam-resistant breast cancer cell lines. Growth of parental MCF7, MDA-MB-415, T47D, and ZR-75-1 and of their Tam-resistant counterpart generated for the present investigation. The growth of parental cells was assessed both in the absence or in the presence of 5 µM Tam; every Tam-resistant subline was grown exclusively in the presence of 5 µM Tam. Data represent the mean ± SD of three independent experiments. Statistical significance was assessed by ANOVA followed by the Tukey *post-hoc* test; *: $p < 0.05$; ***: $p < 0.001$.
